# Telemedicine Interventions as an Attempt to Improve the Mental Health of Populations during the COVID-19 Pandemic—A Narrative Review

**DOI:** 10.3390/ijerph192214945

**Published:** 2022-11-13

**Authors:** Anna Rutkowska

**Affiliations:** Department of Physical Education and Physiotherapy, Opole University of Technology, 45-758 Opole, Poland; a.rutkowska@po.edu.pl

**Keywords:** mental health, telemedicine, COVID-19, well-being, preventive medicine

## Abstract

Published reports indicate the need for psychological interventions and the integration of psychiatric care into crisis management plans in people with mental health issues caused by the COVID-19 pandemic. It seems crucial to identify the root causes of the health-social-economic crisis and identify potential opportunities for widely implemented psychological assistance. This narrative literature review aims to identify the types of interventions deployed as telemedicine-based mental health support and their effectiveness. The PubMed and Web of Science electronic databases were searched. From a total of 48 articles, 46 were analysed after removing duplicates. From these, thirty-seven records were excluded according to the inclusion criteria and nine (eight RCT and one cross-over) were assessed as full texts. The included publications were randomised clinical trials or cross-over studies focused on remote mental support interventions. In all studies, participants represented both sexes and had an average age range of 6–64. Studies included participants from seven countries and the overall number of participants in the included studies was 687. The content of these intervention programmes includes both established psychotherapeutic programmes, as well as new interventions. Remote support was implemented through three approaches: phone/video calls, mobile applications, and internet-based programs. The results of the included studies indicate a higher or equal efficacy of telemedicine interventions compared to traditional forms. The review also revealed a relatively wide range of targeted research groups: from children with social anxiety through to their caregivers; adolescents with neurological disorders; and from college students to adults with psychiatric or orthopaedic disorders. Analysis of the included papers found that telemedicine interventions show promising results as an attempt to improve population mental health during the COVID-19 pandemic.

## 1. Introduction

Coronavirus disease 2019 (COVID-19) was first known to the global public in December 2019, but in March 2020 the World Health Organization (WHO) classified the global spread of severe acute respiratory syndrome coronavirus 2 (SARS-CoV-2) as a pandemic [1]. Since then, governments have employed a variety of preventive strategies, including stay-at-home orders, social distancing, hygiene regimes, and business and assembly lockdowns. While it appears that the interventions have been effective in reducing viral transmission, limited access to sports clubs, gyms and parks has resulted in massive declines in physical activity (PA) [2,3].

Limited outdoor areas and facilities for movement and exercise can foster sedentary behaviour. The health consequences of inactivity have previously been described in detail, and it has been suggested that this contributes to up to 9% of all premature deaths [4], and leads to the development of cardiovascular disease [5,6], hypertension, metabolic syndrome [7], type 2 diabetes, and cancer [8]. Thus, many have noted the dangers of physical inactivity. However, evidence on the effectiveness of intervention strategies for adult sedentary behaviour is still limited [9]. They tend to address sedentary behaviour mainly by focusing on reducing work-related sedentary behaviour, and have been largely based on social-cognitive models of behaviour change (e.g., the Theory of Planned Behaviour), where the effect of improved physical activity is motivated by the achievement of a target outcome, such as improved health (expectancy-value approach) [10]. Hence, two main trends in modifying sedentary behaviour in relation to a physical condition persist: demonstrating the health benefits that can be achieved, as well as pointing out the risks that may follow.

Another class of adverse effects of physical inactivity on the functioning of the human individual is in terms of mental health, as physical activity not only plays a role in general health protection, but is also associated with mental health. The relationship between physical activity and mental health has been described in detail in pre-pandemic studies, yet in this period it has taken on an even greater importance. Physical inactivity is inversely associated with mental health in both men and women [11] and is directly related to psychological distress and lower mental well-being and life satisfaction [12]. Falck et al. reported that high levels of sedentary behaviour are associated with reduced cognitive performance [13]. Moreover, Saunders et al. analysed 22 individual studies from 10 countries, revealing a relationship between sedentary behaviour and depression [14]. Furthermore, the imposition of restrictions on social meetings appears to have had an impact on the deterioration of the psychological condition of many populations [15,16,17,18].

A further research area related to the consequences of pandemics, which has been widely reported recently, concerns the mental condition of societies. In the initial stage, people were most emotionally affected by the direct threat to their lives, alongside people who were suddenly deprived of their resources due to lock-downs. In the following months, the whole of society began to experience long-term effects in the form of safety hazards for people whose professional work was previously safe. During the COVID-19 outbreak, many psychological problems and important mental health consequences including stress, anxiety, depression, frustration, and uncertainty have emerged progressively [19,20]. Likewise, there are concerns regarding the adverse effects of COVID-19 on mental distress, sleep disturbances, lowered mood, sadness, tearfulness, and impaired concentration [21]. Furthermore, it has been reported that psychological reactions to the COVID-19 pandemic can range from panic behaviour or collective hysteria, through feelings of hopelessness and despair, to suicidal behaviour [19,22,23].

As the general population becomes increasingly vulnerable over time, and published reports indicate that psychological interventions and the integration of psychiatric care into crisis management plans are necessary, it seems essential to identify the root causes of the health-socio-economic crisis and to identify potential opportunities for widely implemented psychological support. Remote methods can be useful in cases where direct psychological intervention is difficult to carry out. As mentioned above, the COVID-19 pandemic has had a large impact on the mental health of populations, and given the reduction of the risk of infection from refraining from leaving the house, a remote method may be reasonable.

Reports indicate that, in terms of cognitive behavioural therapy (CBT), many forms of implementation are available. Conventionally, face-to-face and self-help CBT can be used; however, self-help CBT can be further divided into computerised CBT or internet-based cognitive behavioural therapy (iCBT). Self-help therapy, in various forms, can address problems in treatment accessibility, such as the limited availability of clinicians, or patients’ reluctance to participate in treatment in a clinical setting. Self-help CBT programmes have been used to improve depression, anxiety, and insomnia [24]. In the context of internet-based therapies, the support is provided as self-management by the patient or through interventions with the therapist. Therapist-assisted therapies are more effective, although patients have more freedom in self-help therapies [25]. Moreover, the results of videoconference group eHealth interventions on mental health, led by professionals, have also been described. A systematic review of 20 RCTs including 2438 participants across seven countries revealed that live health professional-led group eHealth interventions had a medium effect on reducing anxiety and depression compared with inactive or active controls. Therefore, it is feasible to obtain treatment remotely in case of difficulties in travelling to a medical facility due to the COVID-19 pandemic. Thus, this narrative literature review aims to identify the types of interventions and their effectiveness when deployed as telemedicine-based mental health support.

## 2. Materials and Methods

### 2.1. Electronic Searches

The literature search was conducted on 2 August 2022, using the PubMed database (National Library of Medicine, 8600 Rockville Pike, Bethesda, MD, USA) and Web of Science (Clarivate, 1500 Spring Garden, Philadelphia, PA, USA). To identify studies from a specific research topic, the COVID-19 filters from PubMed Clinical Queries were implemented. Due to the limited amount of research available, no specific publication dates were used. The following search formula was defined for the PubMed database: “((Intervention*) AND (((COVID-19) AND (Psychotherapy OR Behavior Therapy OR Cognitive-Behavioral Therapy)) AND (Telemedicine OR Telehealth OR Teleconsultation OR Teleconference*))) AND (Therapy/Broad[filter])”. For the Web of Science database, the particular searches for the category and topic were done separately, the categories were combined using OR, and the individual topic search phrases were combined using AND. The following formulae were used: WC = (1) Telecommunications; (2) (Psychology, Multidisciplinary); TS = (1) Intervention*; (2) COVID-19; (3) (“Psychotherapy” OR “Behavior Therapy” OR “Cognitive-Behavioral Therapy”); (4) (“Telemedicine” OR “Telehealth” OR “Teleconsultation” OR “Teleconference*”). The electronic search identified 48 overall results, with no additional records from the grey literature search. In this review, it was decided to include only randomised controlled trials (RCTs) and cross-over studies, aimed at interventions to counteract the effects of a pandemic. In order to present a wide range of remote interventions, there was no restriction on types of mental health distress, nor on age or gender of participants. All abstracts of the included studies have been reviewed. After removing two duplicates, 37 records were excluded due to their unrelated topic or wrong study design and nine full-text articles were analysed.

### 2.2. Assessment of Methodological Quality

The studies’ methodological quality was assessed using the PEDro scale [26], which consists of 11 criteria: (1) specified study eligibility criteria, (2) random allocation of patients, (3) concealed allocation, (4) measure of similarity between groups at baseline, (5) patient blinding, (6) therapist blinding, (7) assessor blinding, (8) fewer than 15% dropouts, (9) intention-to-treat analysis, (10) intergroup statistical comparisons, and (11) point measures and variability data. The methodological criteria were scored as follows: yes (1 point), no (0 points), or unclear (0 points). The PEDro score categorises studies by quality as follows: excellent (9–10), good (6–8), fair (4–5), and poor (<3) [27].

## 3. Results

**Figure 1:**
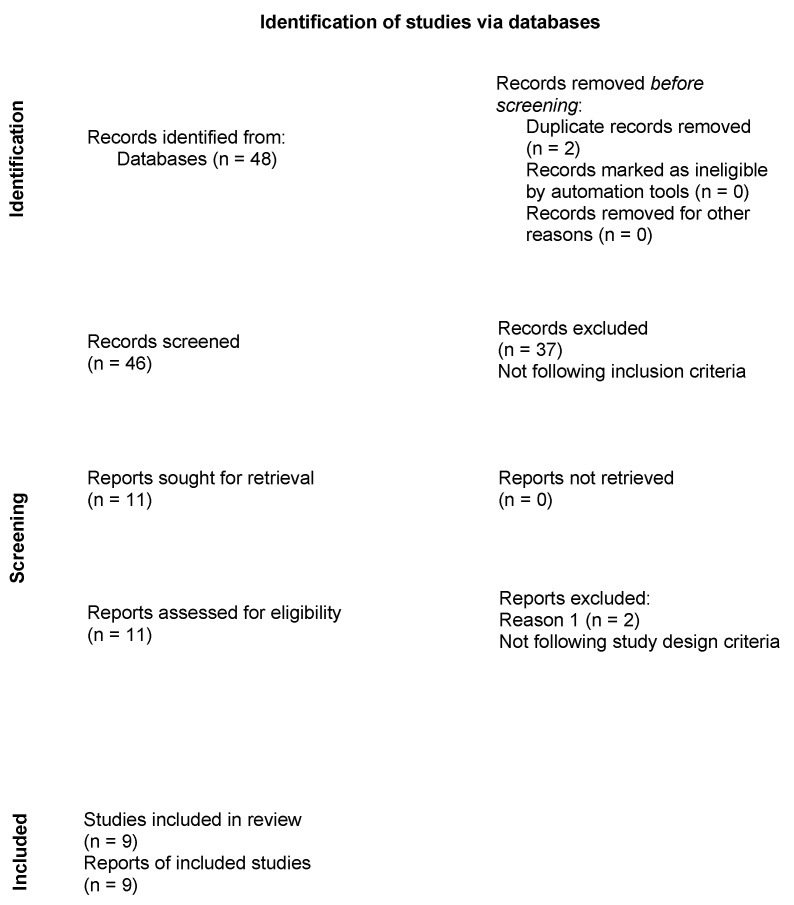
Flow diagram for the study selection process.

The review process presents the PRISMA flowchart (Figure 1).

### 3.1. Characteristics of Included Studies

The included publications were randomised clinical trials or cross-over studies focused on remote mental support interventions. In all studies, participants represented both sexes and were in the average age range of 6–64 years. Studies included participants from seven countries. The overall number of participants in the included studies was 687 (Table 1).

#### 3.1.1. Internet-Based Intervention

Three of the included studies [28,29,30] were internet-based interventions; however, the treatment methods in each study were different. A study by Comer et al. compared the effectiveness of the iCALM Telehealth Program compared with the waitlist of children with social anxiety disorders. The study included 40 children between the ages of 3.0 and 8.9 years and their parents, who were seeking services for early child anxiety. More than half of the caregivers participating in the study were born outside the United States. The families came from diverse financial backgrounds. Families randomly assigned to iCALM participated in a 12-session, manual-based modification of Parent-Child Interaction Therapy (PCIT) for the treatment of early childhood anxiety, provided via the internet. Families were given 16 weeks to complete the therapy. The first four sessions of CALM focus on building a positive and mutually satisfying parent-child relationship. The family works with the therapist to create an individual anxiety hierarchy that provides a roadmap for subsequent progressive exposure sessions. During the following eight sessions, parents are instructed in real-time in guiding their children through increasingly difficult exposures (e.g., approaching an anxiety-provoking situation, such as talking to a stranger or an unfamiliar peer). The primary outcomes measured included: Child Behavior Checklist (CBCL), Child Behavior Questionnaire (CBQ), Child Anxiety Impairment Scale (CAIS), and Depression Anxiety Stress Scale 21 (DASS-21). Analysis of the results showed that iCALM led to significantly greater reductions in children’s anxiety symptoms, children’s anxiety, children’s discomfort, and social impairment related to anxiety, and led to greater improvements in children’s mildness [28]. A study by Rachamim et al. evaluated the feasibility and effectiveness of an internet-based, self-help CBIT (iCBIT) programme compared with the waitlist on global impairment and functioning in young people with tic disorders. The study included 41 participants aged 7–18 years. The primary outcomes consisted of YGTSS, Clinical Global Impression-Improvement Scale (CGI-I) and clinician-rated Global Assessment Scale for Children (CGAS) scales. Secondary outcomes included nine scales assessing anxiety, tic severity, depressive symptoms, and satisfaction. The iCBIT intervention was based on phone calls and teleconferences encouraging attendance to the programme, with the goal of providing both the child and caregiver with specific behavioural and cognitive skills for dealing with tics, and facilitating caregiver support. The purpose of the assistants’ support was to check module completion and adherence, to send a reminder when a participant failed to complete tasks, and to provide support to caregivers. The intervention consisted of nine weekly child-caregiver modules. The average support time was 7.44 min (SD = 1.86) per family per week. Results showed clinically significant reductions in tic severity and improvements in global impairment and function, comparable to face-to-face delivery treatment. Adolescents using iCBIT experienced improvements in self-esteem and comorbidities, and rated the intervention as highly acceptable and satisfying [29]. A study by Wagner et al. evaluated a targeted eHealth intervention, “FoRtitude”, to reduce fear of recurrence using cognitive behavioural skills training and telecoaching. The study included 196 breast cancer survivors, 1–10 years post primary treatment, with moderate to high FoR and familiarity with the internet. However, no detailed socio-demographic data were provided. The primary outcome measured was Fear of Cancer Recurrence Inventory (FoR), while The Concerns About Recurrence Scale (CARS) was employed as a secondary outcome assessment. A multi-step optimisation strategy was used to evaluate three CBT strategies (relaxation, cognitive restructuring, worry practice) vs. an attention control condition (health management content (HMC)). The FoRtitude eHealth website included didactic content, interactive tools, and an interactive text messaging feature provided three times a week. Participants were encouraged to use the FoRtitude site several times a week for 4 weeks. The analysis revealed that the magnitude of reduction in FCRI scores was comparable across CBT and attention control HMC conditions and was predicted by increased self-efficacy [30].

#### 3.1.2. Mobile-Based Intervention

In the two included studies, the remote interventions were based on mobile applications [31,32]. The interventions used a system of sending text messages as well as more advanced features like displaying short videos or audio records. Anthony et al. evaluated the effectiveness of ACT delivered via a mobile phone messaging robot to patients who had their total hip arthroplasty or total knee arthroplasty procedures postponed due to the COVID-19 pandemic, compared to those not receiving messages. The study included 90 adults; however, the sociological-demographic characteristics were not comprehensively presented by the authors. Groups did not differ from any Patient-Reported Outcome Measures (PROM) at enrolment. As outcomes, the authors chose measures of surgical success: PROM both for Physical Health and Mental Health, and Knee or Hip Disability and Osteoarthritis Outcome Score Joint Replacement (H/KOOS JR). The remote intervention was based on sending (or not for the control group) identical text messages to each participant for 14 days, twice a day. The ACT messages were developed in collaboration with a pain psychologist specialising in ACT for chronic pain. The messages used the basic principles of ACT and were designed to help recipients develop better coping skills for managing osteoarticular pain. Comparative analysis showed a 38% improvement in patients in the intervention group and a 17.5% improvement in the control group in the PROM PH. For the HOOS JR and KOOS JR, 24% of the ACT group achieved minimal clinically important differences compared to 2.5% in the control group. The authors concluded that the psychological intervention delivered via text messaging improved physical functioning and prevented deterioration in outcome measures reported by patients who experienced an unexpected surgical delay during the COVID-19 pandemic [31]. Sun et al. evaluated the effect of a mindfulness-based mobile health (mHealth) intervention among young adult students with elevated anxiety and/or depressive symptoms compared to a time- and attention-matched social support-based mHealth control. The study included 114 university students experiencing elevated psychological distress. The primary outcomes measured were the PHQ-9 and GAD-7 scales, while the Mindful Attention Awareness Scale (MAAS) and The Multidimensional Scale of Perceived Social Support (MSPSS) were considered as secondary outcomes. The intervention was based on a 4-week intervention, the “Mindfulness for Growth and Resilience” programme, and included weekly one-hour video conferences via Zoom and a WeChat-based mini-programme that included 20 videos and audio recordings for daily audio-based mindfulness learning. Social support-based mHealth was delivered via Zoom and WeChat as four, weekly one-hour sessions to discuss shared experiences and promote peer support. Analysis of the results showed that mindfulness mHealth had a superior effect on reducing anxiety, although both interventions had a similar effect on improving depressive symptoms. In their conclusion, the authors indicated that a potential combination of self-learning, practice, and group components could significantly reduce the workload of providers [32].

#### 3.1.3. Real-Time Video or Phone Call

In this intervention category, four studies were included [33,34,35,36]. The remote mental support programme concerned different diagnosis groups of participants, both adults and adolescents. The study by Al-Alawi et al. included a comparison of a 6-week-long therapist-guided online therapy course with self-help and internet-based (email-delivered) therapy focusing on COVID-19-induced symptoms of anxiety and depression. The study included 46 participants with an average age of 28.5 (8.7) years. In total, 57% of participants were single, 50% were unemployed, and 96% declared no chronic illness. Preliminary analysis showed that 24% presented symptoms of depression, 33% of anxiety, and 43% of both disorders. The primary outcomes measured were the Patient Health Questionnaire-9 (PHQ-9) and General Anxiety Disorder-7 (GAD-7) scales. The intervention arm received weekly sessions from a trained and licensed psychologist via the Zoom Video Conferencing platform. The initial sessions focused on building a rapport and providing psychological first aid. The subsequent sessions employed principles of CBT and acceptance and commitment therapy (ACT) interventions. As reported, CBT employed the following methods: discussion of automatic thoughts, core beliefs, and schemas; identification of cognitive distortions; and cognitive restructuring. Unique ACT components such as experiential acceptance and willingness, de-fusion, mindfulness training, and encouragement of value-driven living were also applied by therapists, while ensuring adherence to the same orientation throughout the sessions. Participants assigned to the control arm received an automated weekly newsletter with behavioural tips based on CBT and ACT principles, such as focusing on positive cognitive reinforcement, strengthening relationships and mindfulness practices, via email. The authors noticed non-significant differences in the baseline characteristics between both groups. Analyses of the results showed improvements in the scores in both groups, but the GAD-7 scores of participants in the intervention group were significantly more reduced than those of participants in the control group. Moreover, a greater reduction in mean PHQ-9 scores was observed among participants in the intervention group. The authors concluded that therapist-guided online therapy was found to be superior to self-help, internet-based therapy [33]. MacKinnon et al. aimed to evaluate the effect of the Building Emotional Awareness and Mental Health (BEAM) programme on maternal mental health, likewise to determine acceptability and feasibility. The study consisted of 65 mothers, or other primary caregivers who identify as a woman (e.g., grandmother, aunt), of a child aged 18–36 months old, experiencing moderate to severe depression. In total, 76.9% of the total sample were married, with an average age of 33.8 years, 41.5% had undergone previous treatment with psychiatric medication, 27.7% individual counselling, and 15.4% group counselling. The intervention group participated in the BEAM programme and the control group in “treatment as usual”. The BEAM programme, which combines maternal mental health treatment and parenting skills training with clinical peer support and social contact, was used as an intervention. The main goal of the programme was to improve symptoms of depression and promote positive parent-child relationships. The programme was implemented using the Zoom platform and included 10 weekly modules, each consisting of five main components of the programme: expert-led psycho-educational videos, a monitored closed-group online forum, weekly 1 h structured telehealth group sessions, homework (worksheets, reflections, practice exercises, and strategies), and completion of a survey measuring symptoms of depression and parenting stress. The primary outcomes included PHQ-9 and PSI-SF assessment, and the secondary outcomes included GAD-7 and PROMIS self-report measures. Analysis of the pre-post results indicated greater effects on reducing general mental health problems, particularly anxiety and sleep symptoms, among BEAM participants vs. control participants. No significant treatment effects were found for other mental health symptoms, parenting problems, positive coping, or child behaviour [34]. A study by Policarpo et al. assessed the impact of a remote dietary intervention in a cohort of non-alcoholic fatty liver disease patients living with human immunodeficiency virus (HIV). The study included 55 patients with an average age of 54.2 (10.8) years. Baseline characteristics revealed that 29% of patients were in the normal body mass index (BMI) category, 44% were overweight, and 27% were obese. Patients were randomised to general dietary recommendations or to a structured dietary intervention with remote consultation. A video and/or phone interview aimed to characterise eating habits and lifestyle changes and evaluate stress and depression. However, the details of the intervention and the duration of the consultation were not indicated in the study. Analysis of the results showed the impact of the lockdown on the weight, eating habits, physical activity, and mood of the group of patients with NAFLD-HIV; however, maintenance of the dietary intervention using telemedicine can mitigate the change in eating habits and physical activity patterns, preventing significant weight gain [35]. Prato et al. compared the effectiveness of online remote behaviour therapy with face-to-face therapy in young people with Tourette syndrome (TS). The study included 40 patients aged 9–16 years, all affected by TS, with a mean age of tic onset of 5.8 years. The most common comorbid psychiatric disorder was obsessive-compulsive disorder (60%). The Yale Global Tic Severity Scale (YGTSS) and Premonitory Urge for Tics Scale (PUTS) were used to assess the severity of motor and phonic tics. In addition, patients underwent a clinical evaluation conducted by a paediatric neuropsychiatrist. Behavioural therapy was provided in the form of habit reversal or exposure training with response prevention over eight weekly sessions. Participants were randomly assigned to the face-to-face or online remote groups. Remote sessions were conducted via the Skype platform, where a therapist at the clinic connected with patients residing at home. Analysis of the results showed that both forms of BT were equally effective in reducing the severity of tics, obsessive-compulsive symptoms, and anxiety symptoms, as assessed by the neuropsychological findings. However, online remote behaviour therapy was more effective for reducing depressive symptoms than face-to-face behaviour therapy [36].

### 3.2. Assessment of Methodological Quality

Analysis of the quality of the included studies showed that three studies [29,32,34], presented excellent quality, four studies, [28,30,33,36], were of good quality, while two studies, [31,35], demonstrated fair quality. Table 2 shows the PEDro scores of the included studies.

## 4. Discussion

The COVID-19 pandemic has disrupted traditional face-to-face mental health interventions, resulting in the widespread implementation of remote services via telephone, video conferencing, or interactive interned-based programmes. This narrative review provided featured studies with a wide range of types of mental support interventions. The content of these intervention programmes varied markedly. The authors evaluated the effectiveness of both strictly psychotherapeutic programmes like CBT, ACT, PCIT, and BEAM, as well as proprietary supportive interventions. This review also demonstrated a relatively wide range of target study groups: from children with social anxiety to their caregivers, adolescents with neurological disorders, and from students to adults with psychological or orthopaedic disorders. Support accessibility ranged from 14 days to 16 weeks, with different weekly frequencies. In the included studies, anxiety and depression symptom outcomes were assessed most frequently. All of the included studies showed an improvement in the mental condition of the participants after applying the intervention. A review of the results revealed that two papers showed evidence that remote programs were characterised by higher adherence compared to traditional programs. Moreover, four papers indicated that reductions in anxiety symptoms were greater in the remote groups, and two indicated that distance interventions were characterised by better effects to reduce depressive symptoms. It was also noted that the programme is likely to be self-administered and/or managed by health care professionals, while the programme’s content and delivery approach varies depending on patient groups. Thus, it seems that telemedicine interventions have the potential to improve the mental health of populations throughout the COVID-19 pandemic, although it is worth stressing that they may also represent the only possible support approach at times.

Among the undisputed advantages of remote interventions, the most important is their accessibility, particularly in rural areas [37]. Secondly, telehealth demonstrates its multiplicity among clinical disciplines, which benefits the comprehensiveness of medical management. Third, the results show that the telehealth approach may be more effective and efficient, being less time-consuming, more convenient, and patients being more likely to follow the treatment [38,39]. Moreover, the possibility of self-realised therapy in a home setting, regardless of whether it is conducted synchronously or asynchronously, can also be beneficial in terms of individual barriers related to appearing in a mental health facility, or inability to attend due to the quarantine. Adherence to remote programmes has previously been shown to be at a high level. On the other hand, it has been shown that clinician-guided delivery is more acceptable than self-guided intervention [40,41]. Recent systematic reviews have shown that videoconferencing interventions delivered by health care professionals are effective in improving anxiety and depression in adults when delivered live and one-on-one [42,43]. Thus, a therapist’s involvement in telehealth intervention provides better benefits, but has time and cost implications that can limit accessibility. A possible solution is to offer therapist-led eHealth interventions in groups. If effective, group eHealth interventions could increase public access to mental health professionals during and after a pandemic [44].

The results of this review are in line with the findings of previous systematic reviews on the relevance of introducing mental support methods due to the pandemic. Shirotsuki et al., in a descriptive review, reported the effectiveness of iCBT on a population of children with anxiety. The evaluation showed greater efficiency of iCBT than traditional methods. However, they have indicated that the available literature highlights the problem of availability of IT equipment and internet access to include support for broader target groups [24]. Similar findings were reported by Komariah et al. [40]. A systematic review along with a meta-analysis assessed the effectiveness of iCBT in treating depression (6778 patients) and anxiety (6556 patients) among global populations. The meta-analysis of twelve studies revealed a significant effect with a pooled MD of 4.73 (95% CI: 4.55–4.90) in the depression comparison, and a significant pooled MD of 4.84 (95% CI: 3.85–5.83) in the anxiety comparison. However, the results should be considered with caution due to the high heterogeneity (I^2^ = 93% and 95%). High heterogeneity also characterised the systematic review by Currie et al., who evaluated the effectiveness of group eHealth interventions delivered by health professionals on mental health [44]. A medium effect was noted for anxiety reduction compared to inactive interventions or active controls, as well as a medium and small effect for depression reduction compared to inactive and active controls. A recent meta-analysis evaluated the effectiveness of videoconference-delivered CBT (VCBT) compared to control conditions. Sixteen studies with a total of 1745 patients were included, most of which evaluated changes in depressive symptoms. The high heterogeneity may also have influenced the results of the meta-analysis; however, the authors indicated a statistically significant efficacy of VCBT, highlighting the limited number of studies on specific psychiatric and somatic conditions [45]. Moreover, the need for greater research on the effectiveness of remote mental health support methods is widely suggested [46,47,48]. It should be emphasised, however, that there is a continued need to adopt remote mental health services or settings in different countries to strengthen practice and assistance during, as well as after, a global pandemic.

## 5. Conclusions

This review revealed that telemedicine interventions have been implemented as mental health support during the COVID-19 pandemic. The included studies’ intervention groups covered both chronically ill and temporarily mentally affected by the pandemic individuals. Remote support was implemented through three approaches: phone/video calls, mobile applications, and internet-based programs. The results of the included studies indicate a higher or equal efficacy of telemedicine interventions compared to traditional forms. The therapeutic tools consisted of psychotherapy sessions conducted by specialists, or exercises/practices to improve mood. High adherence to remote programmes remains an important conclusion of this review. Analysis of the included papers found that telemedicine interventions show promising results as an attempt to improve population mental health during the COVID-19 pandemic.

## Figures and Tables

**Table 1:** Characteristics of the included studies.

Authors, Year, Study Design	Study Location	Recruitment	Sample Size	Mean Age	Participants Characteristics	Intervention	Frequency	Assessment	Effects
Al-Alawi (2021) et al. RCT	Oman	Recruitment was based on an online survey. The research team contacted eligible participants by email	46 Adults: 36 Female, 10 Male	28.5	Individuals with depression and anxiety. Participants were mostly singles and completed education up to the college level. The majority were self-quarantining.	iCBT	6 sessions	PHQ-9, GAD-7	Online therapist-guided therapy led to a significant reduction in psychological distress. Both GAD-7 and PHG-9 scores in the intervention group were more reduced than in the control group
Anthony (2022) et al. RCT	United States	Recruitment was based on a phone call among patients on the waiting list for surgery	90 Adults: 35.5% Female, 64.5% Male	64	Patients scheduled for ATH or TKA who experienced a surgical delay due to the COVID-19 pandemic. Participants presented minimal to severe levels of anxiety regarding becoming infected with COVID-19	ACT	Twice a day text messages communicating an ACT-based intervention for 14 days	PROM MH, PROM PH, HOOS JR, KOOS JR	An automated mobile phone ACT’S messages can prevent clinically significant decline, and improve physical function and joint-specific PROMs in patients who experience a surgical delay
Comer (2021) et al. RCT	United States	Recruitment was based on a phone call, REDCap survey, and/or videoconferencing among caregivers of children registering for university-affiliated child mental health centre	40 Children: 72.5% Female 27.5% Male	6.2	Children with social anxiety disorder. The majority was a racial minority. Over half of the caregivers were born outside of the United States.	iCALM Telehealth Program	16-weeks remotely delivered treatment	CBCL, CBQ, CAIS, DASS	iCALM led to significantly greater reductions than waitlist in child anxiety symptoms, fear, discomfort, and anxiety-related social impairment, and greater improvements in child soothability.
MacKinnon (2022) et al. RCT	Canada	Recruitment was based on an online advertisement on social media, likewise postings by community partner agencies via electronic mailing lists or public announcements.	65 Adults: 100% Female	33.8	Mothers of toddlers with moderate-to-severe depression	BEAM	10-week app-based digital intervention	PHQ-9, PSI-SF, GAD-7, PROMIS	The BEAM group revealed greater reductions in overall mental health problems, specifically anxiety and sleep symptoms, compared to control group.
Policarpo (2021) et al. RCT	Portugal	Recruitment was conducted among patients from an outpatient clinic	55 Adults: 15 Females 40 Males	54.2	Patients diagnosed with NAFLD-HIV, receiving dietary recommendations or for structured dietary intervention	Video and/or telephone interview	3-months follow-up	Body composition, dietary and lifestyle behaviours	The application of remote maintenance of dietary intervention can mitigate the change in eating habits and physical activity pattern, preventing significant weight gain.
Prato (2022) et al. RCT	Italy	Recruitment was conducted among patients from an outpatient clinic	40 Youths: 4 Females 36 Males	13.5	Youth patients with Tourette Syndrome	Videoconferencing behaviour therapy	8 weekly sessions	YGTSS, PUTS, WISC-IV, YGTSS, CY-BOCS, MASC, PUTS, CDI, CPRS	Online remote and face-to-face behaviour therapy are equally effective in the treatment of severity of tics, obsessive-compulsive symptoms, and anxiety symptoms. Yet remote therapy was more effective for reducing depressive symptoms
Rachamim (2022) et al. cross-over study	Israel	Recruitment was based on a phone call with child’s parent, among proteges of children’s hospital, medical centre and local association	41 Youths: 12 Females 29 Males	11.2	Children and adolescents with tic disorders	iCBIT	9-week program	YGTSS, CGI-I, CGAS	Adolescents using iCBIT experienced improvements in self-esteem and comorbidities. A high retention rate (92%) was noted.
Sun (2022) et al. RCT	China	Recruitment was based on online WeChat-based flyers and websites targeting college students	114 Students: 73% Female 27% Male	22.2	University students with depression and anxiety symptoms. The majority were undergraduate females.	Mindfulness-based mHealth	4-week program	PHQ-9, GAD-7	Mindfulness mHealth intervention had a superior effect on anxiety compared to social support mHealth, while both conditions had a similar effect on improved depression.
Wagner (2021) et al. RCT	United States	Recruitment was conducted among patients from a cancer centre and three cancer institutes	196 Adults	54.7	Breast cancer survivors. Most were non-hispanic whites, employed ≥32 h/wk, with various histories of oncology treatment.	iCBT	4-week program	FoR, CARS	The results indicate that CBT and HMC made similar contributions to reducing FCRI scores, and Telecoaching programme was associated with lower absenteeism and higher website use.

ACT = Acceptance and commitment therapy; BEAM = Building Emotional Awareness and Mental Health; CAIS = Child Anxiety Impairment Scale; CARS = Concerns About Recurrence Scale; CBCL = Child Behavior Checklist; CBQ = Child Behavior Questionnaire; CDI = Child Depression Inventory; CGASC = Clinician-rated Global Assessment Scale for Children; CGI-I = Clinical Global Impression-Improvement Scale; CPRS = Conners’ Parent Rating Scale; CY-BOCS = Children’s Yale-Brown Obsessive-Compulsive Scale for Children; DASS = Depression, Anxiety, Stress Scale; FoR = Fear of Cancer Recurrence Inventory; GAD = General Anxiety Disorders; HIV = Human Immunodeficiency Virus; HOOS JR = Hip Disability and Osteoarthritis Outcome Score Joint Replacement; iCBT = Internet-Based Cognitive Behavioral Therapy; KOOS JR = Knee Disability and Osteoarthritis Outcome Score Joint Replacement; MASC = Multidimensional Anxiety Scale for Children; NAFLD = Non-Alcoholic Fatty Liver Disease; PHQ = Patient Health Questionnaire-9; PROM MH = Patient-Reported Outcome Measure Mental Health; PROM PH = Patient-Reported Outcome Measure Physical Health; PROMIS = Patient-Reported Outcomes Measurement Information System; PSI-SF = Parenting Stress Index—Short Form; PUTS = Premonitory Urge for Tics Scale; PUTS = Premonitory Urge for Tic Scale; THP = Total Hip Arthroplasty; TKA = Total Knee Arthroplasty; WISC-IV = Wechsler Intelligence Scale for Children; YGTSS = Yale Global Tic Severity Rating Scale; YGTSS = Yale Global Tic Severity Scale.

**Table 2:** The PEDro scale score of the included studies.

Article	Eligibility Criteria	Random Allocation	Concealed Allocation	Baseline Comparability	Blind Subjects	Blind Therapist	Blind Assessors	Adequate Follow-Up	Intention-to-Treat Analysis	Between-Group Comparison	Point Estimate and Variability	Total
Al-Alawi et al.	Y	Y	U	Y	N	N	Y	Y	N	Y	Y	7
Anthony et al.	U	Y	U	Y	N	U	U	Y	N	Y	Y	5
Comer et al.	Y	Y	U	Y	N	U	U	Y	Y	Y	Y	7
MacKinnon et al.	Y	Y	Y	Y	N	U	Y	Y	Y	Y	Y	9
Policarpo et al.	Y	Y	U	Y	N	N	U	U	N	Y	Y	5
Prato et al.	Y	Y	U	Y	N	N	Y	Y	N	Y	Y	7
Rachamim et al.	Y	Y	Y	Y	N	N	Y	Y	Y	Y	Y	9
Sun et al.	Y	Y	Y	Y	Y	N	Y	Y	Y	Y	Y	10
Wagner et al.	Y	Y	U	Y	Y	Y	U	Y	Y	Y	N	8

Y = Yes, N = No; U = unclear.

## Data Availability

Not applicable.
